# Effectiveness of palliative care interventions offering social support to people with life‐limiting illness—A systematic review

**DOI:** 10.1111/ecc.12837

**Published:** 2018-03-24

**Authors:** N. Bradley, M. Lloyd‐Williams, C. Dowrick

**Affiliations:** ^1^ Academic Palliative and Supportive Care Studies Group Institute of Psychology Health and Society University of Liverpool Liverpool UK

**Keywords:** day care, group interventions, palliative care, psychosocial care, Social support, systematic review

## Abstract

Individuals managing the challenges of life‐limiting illness require adequate social support to maintain quality of life. Qualitative research reports that patients value highly the social support obtained in palliative care interventions such as day care and group therapies. This systematic review aims to summarise existing quantitative evidence on palliative care interventions that facilitate social support. Research literature was systematically searched using electronic databases and key journals. Searches returned a total of 6,247 unique titles of which sixteen were eligible for inclusion. Interventions include group therapies, group practical interventions and palliative day care. Outcome measures and study designs were heterogeneous. Only one study used a validated outcome measure of social support. Benefits were influenced by participant characteristics such as baseline distress. Partial economic evaluation was attempted by two studies. Methodological challenges include attrition and use of outcome measures that were insensitive to change. Statistically significant results were reported in psychological and physical domains. Evidence is limited due to methodological issues and a scarcity of quantitative research, particularly regarding long‐term benefits and cost‐effectiveness. Interventions may be more beneficial to some groups than others.

## 1 INTRODUCTION

Social well‐being is long recognised as a component of optimum health (World Health Organisation, 1946). Social support is a resource gained through interpersonal interactions that may comprise emotional support, companionship, information/advice and tangible assistance (Uchino, 2004). Adequate social support is necessary for both physical and mental health and can protect against the negative outcomes of long‐term stress (Hostinar, 2015). Inadequate support confers increased mortality risk comparable with high‐profile risk factors such as smoking and obesity (Holt‐Lunstad, Smith, & Layton, 2010). Social isolation is associated with reduced well‐being and increased depression (Golden et al., 2009), cognitive decline (Gow, Pattie, Whiteman, Whalley, & Deary, 2007), increased pain intensity (Lopez‐Martinez, Esteve‐Zarazaga, & Ramirez‐Maestre, 2008) and mortality (Holt‐Lunstad, Smith, Baker, Harris, & Stephenson, 2015).

Palliative care aims to improve quality of life for patients with life‐limiting illness and their families (Hui et al., 2013), providing support for patients to live as actively as possible (World Health Organisation, 2002). The social world of an individual has potential to contribute to, or alleviate, suffering in life‐limiting illness (Garcia‐Rueda, Valcarcel, Saracibar‐Razquin, & Solabarrieta, 2016). Pain, fatigue and other symptoms can limit opportunities to engage with others, so that declining physical function is paralleled by increasing social restriction (Lloyd, Kendall, Starr, & Murray, 2016). Friends and family members may struggle to accept the diagnosis and feel unable to relate to patient experiences (Wilson & Luker, 2006). Social relationships may also be limited by stigma around illness and death (Garcia‐Rueda et al., 2016).

However, social support is associated with better outcomes in advanced cancer (Applebaum et al., 2014) and other incurable diseases (Tomaka, Thompson, & Palacios, 2006). Obtaining emotional support from others, a common coping mechanism in advanced cancer, is associated with better quality of life and reduced anxiety and depression (Nipp et al., 2016). People coping with the existential challenges of approaching death can find interpersonal relationships to be a key component of experiencing meaning in life (Haug, Danbolt, Kvigne, & DeMartinis, 2016), and social comparisons with people in a similar situation may be helpful when trying to establish a new sense of normality in the presence of advanced illness (Lobb et al., 2013). Therefore, psychological stressors that accompany life‐limiting illness might be alleviated through social support (Crunkilton & Rubins, 2009). Patients and referring clinicians agree that additional social support is helpful (Bradley, Frizelle, & Johnson, 2010, 2011); thus, informal relationships cultivated in palliative care are valuable to stakeholders, but appear underacknowledged by research (Wilson & Luker, 2006).

A broad range of palliative services can facilitate social support—including home visits or remote support delivered via telephone or Internet. However, opportunities to get out of the house and engage with others in a dedicated environment are thought to be beneficial for well‐being, by relieving both physical and psychosocial isolation (Bradley et al., 2010; Stevens, Martin, & White, 2011). Palliative day care offers psychosocial support alongside clinical services—patients in the UK are motivated to attend palliative day care because of potential gains in social support (Goodwin, Higginson, Myers, Douglas, & Normand, 2002; Kernohan, Hasson, Hutchinson, & Cochrane, 2006). Peer relationships developed in face‐to‐face group therapies or support groups are also highly valued by patients (Taylor‐Ford, 2014). Thus, this review focuses on solely on interventions taking place outside of the home.

Reviewers have noted a scarcity of quantitative evidence in the evaluation of palliative care (Aoun & Nekolaichuk, 2014), particularly so for interventions with social goals that can be challenging to define and measure (Bradley et al., 2010; Stevens et al., 2011). The evidence base for economic evaluation is poorly developed (Gardiner, Ingleton, Ryan, Ward, & Gott, 2016), yet it is vital to demonstrate effectiveness and make pragmatic decisions about resource allocation in the context of an ageing population and increasing healthcare demands (Woodthorpe & Foster, 2016). There is moderate evidence that complex interventions can improve quality of life in palliative care, but it is unclear which components of these interventions confer benefit (Catania et al., 2015). The research above suggests that provision of social support may be an influential component of psychosocial interventions in palliative care.

This systematic review aims to summarise available evidence on the effectiveness and cost‐effectiveness of palliative care interventions that facilitate social support, by including studies using at least one quantifiable, validated patient outcome measure.

## 2 METHOD

The review was conducted according to PRISMA guidelines (Moher, Liberati, Tetzlaff, & Altman, 2009). A review protocol was not registered. This review is part of an ongoing project funded by the Economic & Social Research Council. The primary reviewer (NB) conducted searches, data extraction and quality appraisal. A representative sample of 10% of identified titles and papers was independently checked by a second reviewer (MLW) to reduce risk of bias. Any discrepancies during the process were discussed with the entire team (NB, MLW & CFD).

### 2.1 Search strategy

A search string was developed from the PICOS (population, intervention, comparison, outcome and study design) model (Centre for Reviews and Dissemination, 2009) without limitation on comparison condition or study design.

(palliative OR “end of life” OR “advanced cancer” OR terminal OR incurable OR “life‐limiting” OR “life‐threatening”) AND (social* OR communit* OR outreach OR respite OR daycare OR “day care” OR “day centre” OR “day service*” OR “day setting*” outpatient OR group OR service* OR engagement OR support* OR ambulatory) AND (psycholog* OR Psychosocial OR emotional OR wellbeing OR well‐being OR isolation OR loneliness OR quality OR qol OR qaly OR health OR economic* OR benefit* OR cost* OR value OR effect* OR efficien* OR outcome* OR impact OR mortality OR function* OR “service use” OR “resource use”)

The literature was searched up to 30 January 2017 using electronic databases of AMED, CENTRAL, CINAHL Plus, EconLit, PsycINFO, PubMed (including MEDLINE), Social Care Online and Web of Science. MeSH terms for palliative care were used in CENTRAL, NHS EED and PubMed. Key journals were searched using Scopus, and recent volumes were additionally hand searched. Grey literature was included using Google Scholar and Open Grey. The reference lists and citations of all included studies were manually searched.

### 2.2 Inclusion criteria

Palliative care interventions were considered to offer opportunities for social support if they facilitated face‐to‐face interactions with other people, outside of the individual's home. Participants are adult outpatients (at least 18 years old and currently living in the community) with a diagnosis of life‐limiting (incurable) illness, including but not limited to cancer. Group activities, structured group interventions and settings such as palliative day care were included. Publications were included that reported at least one validated, quantifiable patient outcome measure, in any domain; including perceived social support, quality of life, psychological distress and symptoms. Mixed methods papers were included. Only English language papers were included due to resource limitations. There was no other restriction on comparators or study design.

### 2.3 Exclusion criteria

Interventions for children, long‐term care residents or palliative inpatients were not included in this review, as issues of social well‐being are expected to differ in these populations. Interventions consisting entirely of clinical appointments, individual tasks or home‐based activity were excluded. Publications were excluded that were purely descriptive or used only qualitative evaluation.

### 2.4 Quality appraisal

The quality of included papers was appraised using a structured checklist approach designed for disparate data (Hawker, Payne, Kerr, Hardey, & Powell, 2002). The flexibility of this checklist in assessing diverse study designs was considered advantageous for this review. Quality assessment considered nine domains: abstract, introduction, method, sampling, analysis, ethics and bias, results, transferability and implications. Each domain was scored out of 4, with higher scores indicating better quality, giving a maximum score of 36.

### 2.5 Data extraction and synthesis

Data were extracted summarising: study design, sample characteristics, outcome measures, results and methodological difficulties reported. This included results of subsequent analysis and additional publications from the same study. A meta‐analysis was not appropriate due to very high heterogeneity of included studies; thus, results are presented as a narrative summary.

## 3 RESULTS

**Figure 1:**
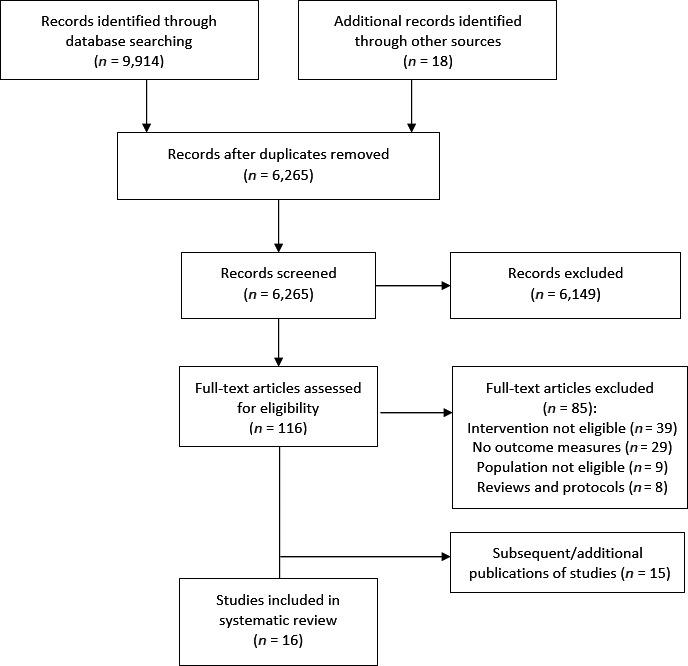
Article selection process for this review. From Moher et al. (2009)

Searches returned 6,247 papers after removal of 3,651 duplicates. Eighteen papers were identified from additional sources (hand searching reference lists, citation checking and key journals). A total of 6,149 papers were excluded based on title and abstract, and the vast majority of these were not relevant to this topic. A total of 116 papers were read in full and assessed for eligibility. Sixteen studies met the criteria and are included in this review (PRISMA flow chart—Figure 1).

### 3.1 Study characteristics

Studies were required to employ quantitative methods, of which three utilised mixed methods. Included studies were from the USA (*n *= 5), UK (*n *= 4), Australia (*n *= 2), Canada (*n *= 2), Ireland (*n *= 1), Poland (*n *= 1) and Sweden (*n *= 1). Participant allocation was frequently randomised (*n *= 10), with other studies designed as non‐randomised comparative studies (*n *= 4) or as pilot studies without a comparator group (*n *= 2). See Table 1 for included studies.

**Table 1:** Details of included studies (*n *= 16)

Study	Quality	Country	Method	Intervention	Population	Study period (attrition)	QOL outcomes	Other outcomes	Subsequent reports
Clark et al. (2013)	31	USA	Randomised controlled trial	Multidisciplinary group intervention	Advanced cancer outpatients *n *= 129 Mean age: 59.3	27 weeks (14.7%)	FACT‐G. Intervention group had higher QOL at week 4 (*p* = .02), significance lost by week 27 (*p* = .88)	FACT‐SWB, POMS, caregiver QOL No significant difference found	Gender differences in response at week 4 (Lapid et al., 2013) After 1 year, participants over 65 (*n *= 16) had longer lasting benefit than participants under 65 (*n *= 38;Chock et al., 2013)
Classen et al. (2001)	30	USA	Randomised prospective study	Supportive‐expressive group therapy	Advanced breast cancer outpatients *n *= 125 Mean age: 53.4	1 year (18.4%)	N/A	POMS, IES Improvement in impact of events scale (*p* = −.03) Secondary analysis removed death‐proximal participants (*n *= 10): statistical difference in mood disturbance (*p* = .02) and greater significance in IES Scale (*p* = .01)	Intervention reduced pain experience (*p* = .001; Butler et al., 2009), improved emotional self‐efficacy (*p* = .55) and emotional suppression (*p* = .01; Giese‐Davis et al., 2002) No survival difference after 14 years, 86% mortality (Spiegel et al., 2007) See also: emotion regulation and diurnal cortisol slope (Giese‐Davis et al., 2006); depression and survival (Giese‐Davis et al., 2011
Edelman, Bell, and Kidman (1999)	30	Australia	Randomised prospective study	Group cognitive behaviour therapy	Advanced breast cancer outpatients *n *= 92 Mean age: 50	6 months (31.5%)	N/A	POMS, Coppersmith self‐esteem inventory Improvements in mood disturbance (*p* = .036) and self‐esteem (*p* = .048), statistical significance lost by 6 months	No survival difference after 5 years, 70.2% mortality (Edelman, Lemon, Bell, & Kidman, 1999)
Edmonds et al. (1999)	31	Canada	Randomised prospective study	Long‐term therapeutic support group	Advanced breast cancer outpatients *n *= 66 Mean age: 50.7	14 months (42.4%)	FLIC. No statistically significant result.	DUFSS, POMS‐SF, MAC, rationality/defensiveness, social desirability Only significant difference in MAC helplessness scale (*p* = .05)	No survival difference after 5 years (*p* = .35) (Cunningham et al., 1998)
P. J. Goodwin et al. (2001)	32	Canada	Randomised prospective study	Supportive‐expressive group therapy	Advanced breast cancer outpatients *n *= 235 Mean age: 50.2	1 year (37.4%)	EORTC QLQ‐C3O. No statistically significant result.	POMS, pain and suffering scale Improvements in mood disturbance (*p* = .02) and pain experience (*p* = .04) after one year Participants with higher baseline distress benefited more from intervention	Cost‐minimisation analysis using resource use and patient outcomes, no significant result (Lemieux et al., 2006) Quality of life analysis reported separately (Bordeleau et al., 2003) See also: report on recruitment and enrolment (Goodwin et al., 2000)
D. Goodwin et al. (2003)	29	UK	Non‐randomised prospective comparative study	Palliative day care	New referrals to five day care centres (mixed diagnoses—96% cancer) *n *= 149 Mean age: 66.2	12–15 weeks (57%)	MQOL. No statistically significant result.	POS. Better pain control at “baseline” (*p* = .065) Better symptom control at 6–8 weeks (*p* = .053), lost by 12–15 weeks	Cost‐effectiveness study using resource use, limited evidence available, inconclusive result (Douglas et al., 2003)
Higginson et al. (2010)	31	UK	Quasi‐experimental prospective comparative study	Palliative day care	New referrals to palliative care (mixed diagnoses—87% cancer) *n *= 132 Mean age: 71.8	12–15 weeks (55.3%)	EQ‐5D VAS. No statistically significant result.	HHI, POS, healthcare usage. Increased hope (*p* = .007) at 6–8 weeks Reduced use of therapeutic healthcare services (*p* = .003)	Increases to hope lost by 12–15 weeks (*p* = .51), but low n due to attrition (Guy, Higginson, & Amesbury, 2011)
Kilonzo et al. (2015)	25	Ireland	Reports on implementation of PROMs (no control)	Palliative day care	Attendees of day care centre (mixed diagnoses—68% cancer) *n *= 102 Mean age: 69	8 weeks (66.7%)	MQOL. 56% of participants improved over 8 weeks	ESAS, EFAT, PCPSS. Symptoms improved in 70.6% of participants Functional status improved in 53% of participants Problem severity improved in 58% of participants	N/A
Kissane et al. (2007)	28	Australia	Randomised prospective study	Supportive‐expressive group therapy	Advanced breast cancer outpatients *n *= 163 Mean age: 51.7	2 years (47.4%)	EORTC QLQ‐C30. Improvement in social functioning subscale (*p* = .03)	MILP, IES, M‐MAC. Reduced depression (*p* = .002), intrusive thoughts (*p* = .04) and helplessness (*p* = .03) at 6 months	Qualitative report on the experience of SEGT (Kissane et al., 2004)
Leppert et al. (2014)	31	Poland	Prospective comparative study across three settings (day care, home care and inpatient unit)	Palliative day care	All advanced cancer patients *n *= 150 Mean age: 67.3	7 days (14%)	EORTC QLQ‐C15‐PAL. All groups improved, significantly higher in day care than other groups (*p* < .0001)	ESAS, KPS. Day care group showed improved well‐being (*p* = .002), fatigue (*p* = .011) and appetite (*p* = .033) Day care group has better performance status at baseline (*p* < .001)	N/A
D. K. Miller et al. (2005)	33	USA	Randomised prospective study	Supportive‐affective group therapy	Outpatients with mixed diagnoses *n *= 69 Mean age: 61.3	1 year (26.1%)	N/A	BDI, SSAI, spiritual well‐being, death distress. Reduced meaninglessness (*p* = .09) Secondary anaylsis removed non‐compliant ppts (*n *= 7): reduced depression (*p* = .04) and improved spiritual well‐being (*p* = .054)	N/A
Roulston et al. (2012)	31	UK (NI)	Pilot study of intervention (no control)	Condition‐specific multidisciplinary group intervention	Advanced lung cancer outpatients *n *= 5 Mean: 63.4	4 weeks (0)	EQ‐5D. Remained constant.	ECOG, EQ‐VAS, HADS. Participants reported improved overall health, anxiety and depression.	N/A
Rummans et al. (2006)	31	USA	Randomised controlled trial	Multidisciplinary group intervention	Advanced cancer outpatients *n *= 103	27 weeks (20.4%)	LASAs. Significant difference between groups at week 4 (*p* = .047), lost by week 27.	POMS‐SF, FACT‐SWB, SDS. No significant difference in overall scales. POMS subscale differences in tension/anxiety (*p* = .42) and confusion/bewilderment (*p* = .014). LASAs subscale differences including social activity (*p* < .0001) and social support (*p* = .001)	Intervention effective at all time points for participants over 65 years (Lapid et al., 2013) Relationship between caregiver age, caregiver QOL and patient QOL (Shahi et al., 2014) See also reports on role of exercise (Cheville et al., 2010)and social worker (Miller et al., 2007)
Spiegel et al. (1981)	26	USA	Randomised prospective study	Long‐term therapeutic support group	Advanced breast cancer outpatients *n *= 58 Mean age: 54.4	1 year (48.3%)	N/A	POMS, HLC, maladaptive coping. Total mood disturbance significantly reduced (*p* < .01), less maladaptive coping (*p* < .01)	Survival difference after 10 years (*p* < .0001) Survival differences independent of clinical treatment (Kogon, Biswas, Pearl, Carlson, & Spiegel, 1997)
Sviden et al. (2009)	26	Sweden	Prospective study with matched comparison group	Palliative day care	Advanced cancer outpatients *n *= 48 Mode age: 51–70	5 weeks (27.1%)	EORTC QLQ‐30. No statistically significant result.	Mood adjective list. Emotional well‐being higher in intervention group but result not statistically significant.	N/A
Tsianakas et al. (2017)	29	UK	Randomised controlled trial	Group walking intervention	Advanced cancer outpatients *n *= 42	24 weeks 45.2%	FACT‐G. No statistically significant result, differences at baseline remained stable	Physical activity, fatigue, stress, anxiety, depression. Only statistically significant difference in level of physical activity	N/A

DUFSS, Duke‐UNC Functional Social Support Questionnaire; EFAT, Edmonton Functional Assessment Tool; EORTC QLQ‐C15‐PAL, European Organisation for Research and Treatment of Cancer Quality of Life Questionnaire—Core 15 Palliative Care; EORTC QLQ‐C30, European Organisation for Research and Treatment of Cancer Quality of Life Questionnaire—Core 30; EQ‐5D, EuroQol 5 Dimensions Questionnaire; EQ‐VAS, EuroQol Visual Analogue Scale; ESAS, Edmonton Symptom Assessment System; FACT‐G, Functional Assessment of Chronic Illness Therapy—General; FACT‐SWB, Functional Assessment of Chronic Illness Therapy—Spiritual Well‐being; FLIC, Functional Living Index for Cancer; HADS, Hospital Anxiety and Depression Scale; HHI, Herth Hope Index; HLC, Health Locus of Control; IES, Impact of Event Scale; KPS, Karnofsky Performance Status; LASAs, Linear Analog Scales of Assessment for QOL; MAC, Mental Adjustment to Cancer Scale; MILP, Monash Interview for Liaison Psychiatry; M‐MAC, Mini‐Mental Adjustment to Cancer Scale; MQOL, McGill Quality of Life Questionnaire; PCPSS, Palliative Care Problem Severity Scale; POMS, Profile of Mood States POMS‐SF, Profile of Mood States‐short form; POS, Palliative Outcome Scale; QOL, Quality of Life; SDS, Symptom Distress Scale; SSAI, Spielberger State Anxiety Inventory.

Most studies (*n *= 12) used only participants with advanced cancer, frequently breast cancer (*n *= 6). Other studies used mixed diagnoses (*n *= 4) with advanced cancer constituting 68%–96% of participants and other diagnoses including lung disease, progressive neurological disease and kidney disease. A wide age range was apparent across studies, from 25 years (Kissane et al., 2007) to 94 years (Leppert, Majkowicz, Forycka, Mess, & Zdun‐Ryzewska, 2014).

Outcome measures used were diverse, with 30 different tools utilised across sixteen studies. Only one study (Edmonds, Lockwood, & Cunningham, 1999) used a validated outcome measure to elicit information on social support—the Duke‐UNC Functional Social Support Questionnaire (DUFSS; Broadhead, Gehlbach, de Gruy, & Kaplan, 1988). Two studies reported on social subscales of outcome measures (Kissane et al., 2007; Rummans et al., 2006). The Profile of Mood States (POMS or POMS‐SF) was the most commonly used outcome measure (*n *= 7). Quality of life was measured with nine different tools by studies reporting this outcome (*n *= 12).

Three categories of interventions were identified, as defined by the original authors. The detail of interventions tends to be poorly described, but there is likely to be considerable overlap in components between these three categories. Group therapeutic interventions (*n *= 7) included supportive‐expressive group psychotherapy and group cognitive–behavioural therapy. Specialist palliative day care (*n *= 5) offers clinical or nursing support in a dedicated environment, alongside group discussion and creative or therapeutic activities. Group practical interventions (*n *= 4) typically include multidisciplinary educational components and group discussion.

### 3.2 Group therapeutic interventions

Group therapy had statistically significant effects on psychological outcomes including mood disturbance (Goodwin et al., 2001; Spiegel, Bloom, & Yalom, 1981), helplessness (Edmonds et al., 1999; Kissane et al., 2007), emotional impact of stressful events (Classen et al., 2001), emotional regulation (Classen et al., 2001), coping (Spiegel et al., 1981), intrusive thoughts and depression (Kissane et al., 2007). Four studies reported survival, with three studies finding no effect (Classen et al., 2001; Edelman, Bell et al., 1999; Edmonds et al., 1999) and one study reporting significant survival effect ten years after the intervention (Spiegel et al., 1981). Two studies reported significant effects on perception of pain (Classen et al., 2001; Goodwin et al., 2001). All studies were randomised, which increases the strength of this evidence.

Participant baseline distress influenced intervention effectiveness (Classen et al., 2001; Goodwin et al., 2001)—more distressed participants benefitted more from the intervention. Edmonds et al. (1999) controlled for baseline psychological differences, intervention compliance and the use of self‐help strategies, reporting a statistically significant result in helplessness only. However, the control group of this study received considerable support (CBT tasks, supportive telephone calls and relaxation exercises). Classen et al. (2001) conducted secondary analysis by removing 10 participants who died within one year of outcome measurement, demonstrating a stronger effect on mood disturbance and emotional impact of stressful events. This approach was supported by unpublished data of a death‐proximal rise in emotional distress in the last year of life. Miller et al. (2005) initially identified non‐significant improvements, but removal of seven non‐compliant participants for secondary analysis showed statistically significant results in depression and spiritual well‐being, suggesting sufficient dose to be necessary.

The Duke‐UNC Functional Social Support Questionnaire was used by Edmonds (Edmonds et al., 1999) and identified no significant change. However, the authors report that the questionnaire prioritised support received from the family unit and therefore appears insensitive to gains in social support occurring as a result of the intervention. Miller et al. (2005) measured baseline social support, stating that it was not expected to be influenced by the intervention (without further elaboration) and controlling for this variable; dropouts from this study had higher baseline social support, suggesting that the group format was not suitable or relevant for these individuals. Edelman, Bell, et al., (1999) suggests social support should be monitored in future studies—warning that minimising participant burden can impede study design through the use of insufficient outcome measures. Poor sensitivity and floor effects of outcome measures were reported (Edmonds et al., 1999; Goodwin et al., 2001).

### 3.3 Palliative day care

Day care was reported to have a statistically significant effect on symptoms (Goodwin, Higginson, Myers, Douglas, & Normand, 2003; Leppert et al., 2014) and hope (Higginson, Gao, Amesbury, & Normand, 2010). Studies with longer study designs found this was not apparent over time, possibly due to attrition and small sample size (Goodwin et al., 2003; Higginson et al., 2010). The majority of participants in an observational study benefitted from 8 weeks of day care (Kilonzo, Lucey, & Twomey, 2015), but attrition makes it difficult to establish whether the intervention was beneficial for non‐completers. Improved symptomology reported by Leppert et al (2014) could have been influenced by baseline differences in physical activity and symptom burden.

Methodological difficulties included large individual fluctuations over time (Sviden, Furst, von Koch, & Borell, 2009) and difficulty in obtaining a true baseline prior to day care attendance due to ethical concerns (Goodwin et al., 2003; Sviden et al., 2009). Goodwin et al. (1998), Leppert et al. (2014) and Sviden (2009) suggest using different outcome measures that might be more responsive, less vulnerable to floor effects and better tailored to the goals of psychosocial interventions, including perception of social support.

### 3.4 Group practical interventions

Group interventions may have a temporary effect on quality of life (Clark et al., 2013); however, three studies (Roulston, Bickerstaff, Haynes, Rutherford, & Jones, 2012; Rummans et al., 2006; Tsianakas et al., 2017) reported that quality of life measures remained stable. Social support was not used as an outcome measure. Rummans et al. (2006) identified improvements in social well‐being through quality of life subscales. A pilot study with mixed methods (Roulston et al., 2012) appeared to have positive effects on mood and perceived health; all five participants cited social support as a useful component of the intervention. Tsianakas et al. (2017) reported that questionnaires used were not sufficiently sensitive, and draws from qualitative insights to suggest that a specific social support measure be used in future work.

### 3.5 Economic evaluation

Economic evaluation was attempted by only two of the included studies—Goodwin et al. (2001) on group therapy for women with metastatic breast cancer and Goodwin et al. (1998) on palliative day care—both attempted a cost‐effectiveness study by hypothesising that an effective intervention would reduce participants’ use of other healthcare resources.

Lemieux, Topp, Chappell, Ennis, & Goodwin (2006), reporting on Goodwin et al. (2001), calculated incremental cost‐effectiveness ratios for change in mood and pain outcomes. A significant difference in healthcare resource use between intervention and control group was not identified; however, the difference in resource use appeared to be larger for participants with higher distress at baseline.

Douglas (Douglas, Normand, Higginson, Goodwin, & Myers, 2003), reporting on Goodwin et al. (1998), presented evidence on intervention cost and highlighted challenges of obtaining accurate cost estimates for multidimensional interventions such as day care. Participant health and social care resource use appeared to differ between the intervention group and comparison group—patients accessing palliative day care accessed fewer community services. Conclusions were limited by group size and extent of missing data.

### 3.6 Quality appraisal

The quality appraisal method used in this review (Hawker et al., 2002) was selected to allow for methodological heterogeneity, as evidence‐based practice cannot rely solely on randomised controlled trials in areas of ethical sensitivity and fluctuating or deteriorating health. All studies were rated as fair for quality (more than 23 points out of a maximum of 36) or good (more than 31 points), although this frequently reflected the quality of reporting rather than the study itself. The lowest scoring domain was ethics and bias, reflecting that issues of confidentiality, consent and bias were not sufficiently articulated in the articles. There is some risk of reporting bias at study level, and it is possible that publication bias may have limited the likelihood of null results being identified.

## 4 DISCUSSION

This review systematically examined available quantitative evidence on palliative care interventions that include facilitation of social support outside of the home. Multiple domains and a heterogeneity of outcome measures were apparent across the sixteen studies included. The majority of papers reported psychological well‐being to be improved by the intervention, indicated by improved mood and reduced depression or fewer maladaptive cognition; seven studies reported significant differences, four studies non‐significant improvements, and two reported no change. Twelve papers reported on quality of life outcomes, five of these identified improvements, but this was statistically significant in only two studies. Only one study used a validated measure of social support, and social well‐being subscales were employed within generic measures in other studies. Economic evaluation was attempted by two studies. Consistent with other reviews (Singer et al., 2016), cancer diagnoses dominate the included studies.

Baseline psychological distress influenced intervention effectiveness: more distressed participants reported more benefit. This is in accordance with a meta‐analysis of 61 studies on psychosocial treatment across all cancer stages, which concluded pre‐intervention distress moderates intervention effects, more so than intervention format, setting or dose (Schneider et al., 2010). Baseline social support might influence participant's experience of an intervention and its acceptability (Miller, Chibnall, Videen, & Duckro, 2005). Participant gender (Lapid et al., 2013) and age (Chock et al., 2013) may be relevant, but research is limited. The relevance of caregiver quality of life was highlighted as an important influence on patient quality of life (Clark et al., 2013; Rummans et al., 2006; Shahi et al., 2014).

Survival differences were reported by one study, but not confirmed by three other studies reporting this outcome. This discordance might reflect sociocultural differences between studies and populations, for example, it has become more acceptable for cancer to be discussed in public (Spiegel et al., 2007). It is also possible that the reported survival difference (Spiegel, Kraemer, Bloom, & Gottheil, 1989) is anomalous: the survival curves of the intervention and control groups did not diverge until after notable attrition had taken place, and the 12 participants remaining in the control group by this time differed markedly from regional survival norms for breast cancer (Fox, 1998). Despite ambiguity over survival, psychosocial interventions can influence other clinical outcomes (Temoshok & Wald, 2002). Sufficient dose may be necessary for effectiveness (Miller et al., 2005), supported by survival analysis of the Edmonds study (Cunningham et al., 1998) identifying differences in active engagement with the intervention to be significantly associated with survival. An adjusted meta‐analysis reported that, with sufficient dose, psychosocial and behavioural interventions may prolong survival for at least some patients with cancer (Xia, Tong, & Feng, 2014); interestingly, a subsequent meta‐analysis across cancer populations reported a survival benefit in group interventions, but not individualised interventions (Fu et al., 2016).

There were a number of methodological limitations reported by included studies. Outcome measures frequently lacked sensitivity to change or specificity to expected domains of outcome; many of the patient‐reported outcome measures used in trials of palliative care lack adequate responsiveness in the context of life‐limiting illness (Kearns, Cornally, & Molloy, 2017). Relying solely on randomised controlled trials can be unsuitable for populations with deteriorating health, warranting creativity in study design (Aoun & Nekolaichuk, 2014). However, a sufficient number of time points and length of study period are required to account for individual fluctuations that may obscure results. Ethical considerations must remain at the forefront of palliative care research, but being overly reticent towards participant burden can damage the validity of results, meaning that the valuable contributions made by participants to research are inadvertently reduced. Attrition is to be expected in this population, and empirical studies should plan statistical power accordingly (Stevens et al., 2011). Distinguishing between types of attrition and reporting on reasons for withdrawal can be revealing (Higginson et al., 2013). Selection of outcome measures and other elements of research design should be informed by the experience of other researchers (Gaertner et al., 2016).

### 4.1 Limitations of review

This review has limitations associated with search strategy and eligibility criteria. We used eight electronic databases, supplemented with hand searching of key journals and checking all citations and references. However, only English language articles and grey literature were searched due to resource limitations, introducing a source of bias and limiting the comprehensiveness of this review.

Intervention descriptions were occasionally insufficient to determine whether or not social support was facilitated—we did not include individualised therapeutic tasks completed in a room alongside others without explicit reference to group communication (Imriea & Troop, 2012)—it is therefore possible that relevant interventions were not included. A number of interventions were retrospectively evaluated as having enabled social support by the researchers, but were designed for a different purpose such as self‐management (Roulston et al., 2012). All interventions involved multiple components, so it is possible that benefits such as symptom control would be more appropriately attributed to other components such as clinical input or self‐management skills. The use of mixed methods in research can help to untangle the relationship between components and outcome (Higginson et al., 2013).

The criteria for inclusion in this review were developed from scoping searches, using patient experience of psychosocial palliative care interventions. We focussed explicitly on interventions taking place outside of the home, informed by a qualitative metasynthesis concluding that a change in scenery and getting out of the house were necessary for alleviation of both physical and psychosocial isolation (Bradley et al., 2010). This meant that well‐established examples of community‐based support programmes, for example the Good Neighbourhood Partnership in Ireland (McLoughlin, Rhatigan, et al., 2015) and the Neighbourhood Network in India (Sallnow, Kumar, & Numpeli, 2013), were excluded from the review. However, models of home‐based palliative care (which may or may not include facilitation of social support) are better represented than other settings in the research literature (Brereton et al., 2017), and we are aware of an ongoing systematic review of community‐led support interventions for adults living at home with palliative care needs (Mcloughlin, Furlong, et al., 2015). An additional consideration is the increasing availability of different formats, for example social networking interventions (Owen, Bantum, Pagano, & Stanton, 2017). Given the high prevalence of limited mobility in this population, it would be appropriate for future reviews to compare social support facilitation across different settings and formats.

## 5 CONCLUSION

Responding to the needs of people with life‐limiting illness requires consideration of social well‐being, including their perception of support from others. Psychosocial palliative care services and interventions have been developed that facilitate social support, with some evidence for effectiveness. It is possible that psychological and physical benefits operate via mechanisms of social support. However, social support does not appear to have been used appropriately as an outcome of palliative care interventions. Further research is required to elucidate what forms of social support are most effective to which patients at which time points. It may be illuminating to test different intervention formats and durations or explore individual differences in baseline distress or perceived social support. With increasing financial pressures, it is crucial that economic evaluation takes place alongside testing intervention effectiveness.

This systematic review summarises the quantitative evidence of benefits of social support interventions to people with life‐limiting illness. Existing evidence suggests that patients presenting with high distress are most likely to benefit from interventions facilitating social support. We suggest, based on this review, that clinicians explicitly consider social needs and risk of social isolation as an important domain within holistic care.
